# Investigating Hydrogen Isotope Variation during Heating of *n*-Alkanes under Limited Oxygen Conditions: Implications for Palaeoclimate Reconstruction in Archaeological Settings

**DOI:** 10.3390/molecules26071830

**Published:** 2021-03-24

**Authors:** Rory Connolly, Margarita Jambrina-Enríquez, Antonio V. Herrera-Herrera, Carolina Mallol

**Affiliations:** 1 Instituto Universitario de Bio-Organica Antonio Gonzalez (IUBO), Universidad de La Laguna, 38206 Canary Islands, Spain; rconnoll@ull.es (R.C.); mjambrin@ull.edu.es (M.J.-E.); avherrer@ull.edu.es (A.V.H.-H.); 2 Departmento de Geografia e Historia, Universidad de La Laguna, 38200 Canary Islands, Spain; 3 Departamento de Biología Animal, Edafología y Geología, Facultad de Ciencias, Universidad de La Laguna, 38200 Canary Islands, Spain; 4 Interdisciplinary Center for Archaeology and the Evolution of Human Behaviour (ICArEHB), Universidade do Algarve, Campus de Gambelas, Edificio 1, 8005-139 Faro, Portugal

**Keywords:** *n*-alkanes, compound-specific isotope analysis, hydrogen isotopes, palaeoclimate, archaeology, thermal alteration, combustion

## Abstract

This paper reports on a series of heating experiments that focus on *n*-alkanes extracted from leaf, bark, and xylem tissues of the *Celtis australis* plant. These lipid biomarkers were analysed for their compound-specific hydrogen isotopic composition (δ^2^H_wax_) under limited oxygen conditions at 150, 250, 350, and 450 °C. Our results reveal isotopic variations in wax lipids of different plant organs during short-term low-temperature combustion. We conclude that, in the absence of a detailed characterisation of the depositional environment in advance of sampling, δ^2^H_wax_ values in archaeological or otherwise highly anthropogenic environments should be interpreted cautiously. In addition, we observed that variation in δ^2^H_wax_ of leaves is minimal at temperatures ≤ 350 °C, highlighting the potential for δ^2^H_wax_ in thermally altered combustion substrates to yield palaeoclimate information, which could allow researchers to investigate links between archaeological and climatic records at a high spatial and temporal resolution.

## 1. Introduction

Compound-specific hydrogen isotope analysis of *n*-alkanes (δ^2^H_wax_) derived from terrestrial and aquatic plant waxes has been shown to record plant source water’s isotopic composition and has thus emerged as a robust proxy for palaeohydrological reconstruction [1,2,3,4]. Numerous studies have reported on the wide range of environmental factors which govern hydrogen isotopic fractionation in plant waxes, which include precipitation δ^2^H, precipitation amount, plant type, plant physiology, temperature, aridity, seasonality, and humidity [5,6,7,8]. However, it is notable that despite the recent upsurge in the application of this proxy in archaeological settings, little attention has been afforded to the potential impacts of human activities on δ^2^H_wax_. Anthropogenic combustion features and their associated residues, which are chronologically and geographically ubiquitous in archaeological contexts, particularly stand out as an important source of potential bias and the effects of short-term low temperature (<450 °C) combustion on δ^2^H_wax_ values are not well understood.

Anthropogenic combustion features, or hearths, have been the focus of a considerable body of archaeological research in recent years using an array of high-resolution analytical techniques [9,10,11,12,13]. The processes involved in forming anthropogenic combustion features are complex and subject to a considerable range of culturally and environmentally mediated variables. However, at the most basic level, these features are composed of a thermally altered substrate overlain by a black combustion layer resulting from the deliberate exploitation and management of fire by one or more humans in the past. Experimental studies suggest that the substrate on which the fire is lit (i.e., occupation surface) do not typically exceed temperatures of 300 °C and may provide preferential conditions for the preservation of lipid biomarkers [14,15]. The most rudimentary form of anthropogenic combustion feature is a simple hearth in which combustion is carried out on a horizontal surface. Other common forms include cuvette fires, or pit hearths, in which the combustion is carried out in a bowl-like depression. In some instances, pebbles or flagstones may be placed around the fire in a horizontal or vertical position and act as heat retainers, altering the combustion’s archaeological and sedimentary signature [16,17]. Traditionally, palaeoenvironmental information from combustion features has been derived from analyses of the associated charcoal assemblages [18,19,20,21] or phytoliths [22,23,24,25], both of which are the product of human activities and depend on the preservation of physical plant remains. The application of lipid biomarker analyses, despite being first approached as early as the 1980s [26,27,28] is still not widely employed for the study of combustion features. In recent years, decreasing costs and improved access to instrumental analyses has led to a modest increase in the number of studies that have sought to exploit lipid biomarkers’ potential and their associated compound-specific isotope ratios to investigate both experimentally derived and archaeological datasets. To date, however, most of these studies have focused on questions related to cooking activities and the identification of potential fuel sources [29,30,31,32,33].

Here, we conducted a series of heating experiments to evaluate molecular and isotopic changes to plant-derived wax lipids during short duration combustion events under limited oxygen conditions using leaves, bark and xylem tissues of the *Celtis australis* plant, commonly known as the European nettle tree or Mediterranean hackberry. This species represents a common fuel source in archaeological settings, particularly in sites dating to the Middle Palaeolithic [14,17], and in such instances is likely to be an important source of organic residues in archaeological combustion features. In addition, fine charred particles produced during the combustion of these fuel sources are likely to be locally dispersed in surrounding sediments. We aimed to assess the degree of preservation of climatic signals in δ^2^H_wax_ in anthropogenic combustion features and account for potential biases that could arise due to the mixing of microscopic fine charred particles dispersed in sediments throughout the site.

## 2. Results

### 2.1. Concentration and Molecular Distribution of n-Alkanes

Alkanes ranging from nC_18_–nC_33_ were detected in the leaf samples, and from nC_16_–nC_31_ in the bark samples and xylem samples. We observed a prevalence of odd-over-even *n*-alkanes, which is the typical distribution pattern observed for higher order terrestrial plant waxes [34]. Total *n*-alkane concentration (µg/g dried sample), Carbon Preference Index (CPI_25-33_), which is determined by the ratio of odd over even carbon number molecular chains and can yield information about the biological source and maturity of organic matter [34], and Average Chain Length (ACL_25-33_) [35] have previously been reported elsewhere [36] and are outlined here for comparison in Table 1.

### 2.2. Variation in δ^2^H_wax_ of Thermally Unaltered Reference Samples and Heated Samples of Celtis Australis

Here we measured the hydrogen isotope composition of different plant organs of *Celtis australis* during short-term combustions (1 h) at temperatures of 150, 250, 350, and 450 °C under limited oxygen conditions. This data is presented in Table 2 below. We report only the δ^2^H_wax_ of nC_27_, nC_29_ and nC_31_ because these long-chain alkanes are among the most widely utilised terrestrial plant biomarkers. 

The maximum variation in hydrogen isotope composition observed for each of the sample sets was ~49‰ (nC_27_), ~80‰ (nC_31_), and ~94‰ (nC_31_) for leaves, xylem, and bark, respectively. Based on the nC_31_ homologue, for which isotopic determinations could be made using unaltered reference samples in all three sample sets, leaves are enriched in ^2^H (−128.2‰) by ~65‰ relative to xylem (−193.4‰) and ~23‰ relative to bark (−151.4‰). For nC_29_ and nC_31_, the most common homologues targeted for terrestrial palaeoenvironmental reconstruction, δ^2^H values in leaves was generally minimal at temperatures up to 350 °C, with a maximum variation of ~4 and ~7‰, respectively, ranging from −131 to −135‰ and −128 to −135‰. This is followed by an increasing trend in δ^2^H_wax_ values at 450 °C. This increase likely arises through the interplay of different factors, including kinetic isotope effects and the formation of alkanes from precursor compounds. In xylem samples, there is a decreasing trend toward ^2^H depleted values between 150 and 250 °C of ~19‰ (nC_27_) and ~35‰ (nC_29_) despite an increase in total *n*-alkane concentration in these samples.

Although reliable δ^2^H values were not obtained for the unaltered xylem samples for nC_27 and_ nC_29_, making it difficult to meaningfully compare that sample set, a general decreasing trend is observed for nC_31_ between the reference samples and those heated to 250 °C. It is notable that bark samples followed a similar trend as the leaf samples toward more depleted values at 250 °C, followed by a slight increase in δ^2^H values at 350 °C. Total variation in hydrogen isotope composition of the heated bark samples was in the range of ~57‰ (nC_27_), ~79‰ (nC_29_), and ~94‰ (nC_31_). 

To summarise, δ^2^H values in *n*-alkanes from leaf waxes were only minimally altered up to 350 °C with an increasing trend toward ^2^H enriched values occurring at 450 °C. In contrast, in wood tissues (bark and xylem) the original unaltered δ^2^H signal was lost at 250–350 °C. The observed variations are similar to those described for δ^13^C_wax_ [36].

## 3. Discussion

Variation in δ^2^H values between different plant organs can at least partly be explained by physiological and chemical differences which affect alkane biosynthesis and isotopic fractionation in different plant tissues [37]. Although it has been demonstrated that isotopic fractionation of plant xylem water does not occur during root uptake and transport to the leaf, thereby ensuring xylem carries the isotopic signature of the source water, significant differences in δ^2^H values have been reported between xylem sap, twigs, roots, and wood core [38]. It has also been demonstrated that hydrogen isotopic fractionation is affected by photosynthetic processes, with significant differences in δ^2^H values observed between plant photosynthetic organs and heterotrophic organs [39]. 

It is widely accepted that long-chain *n*-alkanes (nC_27_–nC_33_), derived from epicuticular leaf waxes, represent the dominant source of *n*-alkanes in sediments [40]. However, in archaeological contexts or other anthropogenic settings we should not discount the possibility that other plant organs may represent a source of sedimentary *n*-alkanes. Well preserved charred plant tissues and fragments of microcharcoal, which are not visible at the macroscopic scale of excavation, are frequently observed in soil micromorphological samples collected from archaeological sites [14,41,42,43]. In occupation horizons, these materials may represent fuel residues directly linked to combustion features, or alternatively, they may have been deposited through natural agents. For instance, microcharcoal is commonly registered in palynological datasets and has been utilised to study the frequency of natural fires linked to climate shifts [44,45,46]. Our findings emphasise the need to carefully consider the depositional setting, particularly relating to anthropogenic combustion activities, when interpreting molecular or isotopic information from sedimentary *n*-alkanes in archaeological contexts. The unintentional incorporation of fine charred particles within sediment samples, which has been subjected to temperatures of 450 °C or greater, could lead to misinterpretations of leaf wax δ^2^H values and molecular ratios, such as CPI and ACL, with significant implications for palaeoclimatic reconstruction. The application of complementary techniques, such as soil micromorphology, can identify charred particles’ presence and help eliminate potential biases in the molecular and geochemical datasets. This approach has already effectively been demonstrated at the Middle Palaeolithic site of Abric del Pastor (Alcoy, Spain), where abrupt fluctuations in *n*-alkane distribution and compound-specific carbon and hydrogen isotope values through stratigraphic units IVd and IVc were interpreted as resulting from the presence of microscopic charred fuel residues in the sediment samples [47]. 

On the other hand, our results here demonstrate that the change in δ^2^H values in leaves was generally minimal at temperatures up to 350 °C, with a maximum difference of ~4 and ~7‰ for nC_29_ and nC_31_, respectively, ranging from −131 to −135‰, and −128 to −135‰. This relatively minor ^2^H depletion is despite a dramatic decrease (~79%) in total *n*-alkane concentration within this temperature range, from 459.1 to 97 µg/g. Although the effects of short-term low-temperature combustion on *n*-alkane δ^2^H values have not been widely investigated, our results are broadly consistent with studies elsewhere which observed a maximum difference of ~10‰ in δ^2^H values of a pure *n*-alkane mixture (nC_15_ to n-C_38_) heated at 100–300 °C [48]. However, it is worth noting that study was based on a heating duration of 24 h under oxygen-free conditions. As these do not reflect real conditions in the archaeological record, and anthropogenic combustion processes are not produced in the total absence of oxygen, there is a need for more studies like ours which seek to replicate field conditions in so far as is possible.

The result from our *Celtis australis* leaf samples is potentially significant, as it implies that low-temperature alteration of fossil leaf waxes within sediments (<350 °C) may not eradicate the hydrogen isotope climate signature, opening the door to high-precision onsite palaeoclimate records from lipid biomarkers recovered from archaeological combustion features. Other studies have drawn attention to the potential for thermally altered sedimentary substrates, which underly the black layer of flat combustion features, to yield archaeological information about human activity preceding the combustion event [14]. These sediments, which represent occupation surfaces on which fires were built, do not typically exceed temperatures of 350 °C and may, therefore, be suitable for compound-specific hydrogen isotope analysis. Such an approach would allow for well-dated palaeoclimate records directly associated with human activities and create a new avenue for exploring human responses to climate change at the site-specific scale. A similar approach has recently been applied elsewhere and demonstrated that δ^2^H values of lipid residues preserved in pottery from Late Neolithic levels at Çatalhöyük carried the climatic signature of the North Atlantic abrupt climate event which occurred 8.2 kya [49].

Notwithstanding the relatively small number of samples analysed here, our results provide the initial steps toward establishing boundary conditions for constraining the maximum potential alteration of fossil leaf waxes in thermally altered sediments. Our results suggest that the thermally altered combustion substrates in archaeological contexts could be targeted for hydrogen isotope analysis of individual *n*-alkanes. 

## 4. Materials and Methods

Leaf and branch samples of *Celtis australis* were collected in July 2015 and April 2016 from the area surrounding the Middle Palaeolithic site of El Salt in Alcoy, Spain (726 m asl). All samples were rinsed with distilled water to remove potential contaminants and oven-dried at 60 °C for 24 h at the Archaeological Micromorphology and Biomarker Laboratory (AMBI), Universidad de La Laguna, Tenerife. Leaf (4 g; 1 cm length) and branch (20 g; 2 cm diameter) samples were placed in ceramic crucibles (4.2 cm × 2.5 cm) and covered with Al foil to limit the supply of O_2_ during the heating process. All samples were heated in a muffle furnace with a ramp rate of 26 °C/min for a duration of 1 h to temperatures of 150 °C; 250 °C; 350 °C; 450 °C. Following this, samples remained in the closed furnace to cool overnight before being homogenised into a fine powder using a pestle and mortar.

Following a single cycle in the laboratory glassware washer, all non-volumetric materials were calcined at 450 °C for a duration of 10 h to eliminate potential contaminants. Lipids extraction was carried out with dichloromethane/methanol (DCM:MeOH, 9:1) (10–20 mL) by ultrasonic extraction (3 × 30 min) at controlled temperatures <30 °C. Samples were subsequently centrifuged (3 × 10 min at 4700 rpm) and filtered through annealed glass wool. Solid-phase column chromatography (2 mL column with glass wool, 0.1 g quartz sand (50–70 mesh) and 1 g of activated silica (70–230 mesh) was used to separate the lipid extract into fractions of differing polarity. Alkanes were eluted with ⅜ dead volume (DV) *n*-hexane (DV 1.5 mL, ⅜ DV 562 µL). Samples were subsequently dried under a steady stream of N2 in an Organomation evaporator, internal standard (IS) 5α-androstane (2000 mg/L in DCM, purity ≥99.9%, Sigma–Aldrich, Bellefonte, PA, USA), 8 mg/L was then added. The compounds were analysed by gas chromatography (GC) with a coupled mass-selective detector (GC-Agilent 7890B, MSD Agilent 5977A, Agilent Technologies, Santa Clara, CA, USA) which was equipped with a HP-5MS capillary column ((5%-phenyl)-methylpolysiloxane, length: 30 m, ID: 250 µm, film thickness 0.25 µm). A temperature program with an initial temperature of 70 °C for 2 min and a heating rate of 12 °C/min to 140 °C and a final temperature of 320 °C was applied with a heating rate of 3 °C/min for 3 min, and a total run time of 82.83 min, using a Helium carrier gas (2 mL/min). The multimode injector was held at a split ratio of 5:1 at an initial temperature of 70 °C for 0.85 min and heated to 300 °C at a programmed rate of 720 °C/min. 

Identification of individual compounds was carried out by comparing retention times and mass spectra to those of reference compounds (mix C_8_-C_40_ and 5α-androstane, Supelco) and mass spectral library databases (NIST). Quantification of concentrations was achieved using calibration curves that plot the ratio Area/Area_IS_ versus the reference compounds’ concentration. Correlation coefficients for each sample were higher than 0.995. Concentration is expressed here in terms of µg of individual compound per gram of dry sample (µg gds^−1^).

Compound-specific hydrogen isotope analysis was performed using a Thermo Scientific Isotope Ratio Mass Spectrometer (IRMS) Delta V Advantage. The IRMS was coupled to a GC Trace1310 through a Conflo IV interface with a temperature converter GC Isolink II, with a Trace Gold 5-MS (Thermo Fisher Scientific, Bremen, Germany) fused silica capillary column ((5%-diphenyl)-dimethylpolysiloxane, 30 m length × 0.25 mm i.d., 0.25 μm film thickness). A helium flow rate of 1.5 mL/min was employed for all measurements carried out in triplicate. Data acquisition and processing were carried out using Isodat 3.0 software (Thermo Fisher Scientific, Bremen, Germany). Hydrogen (δ^2^H) isotope values are reported for *n*-alkanes C_29_ and C_31_. Samples were injected using a Programmed Temperature Vaporising injector (PTV) in splitless mode. Temperatures initially increased from 60 to 79 °C (held 30 s, rate 10 °C/min), and then to 325 °C (held 3 min, rate 10 °C/s), and finally to 350 °C (held 3 min, rate 14 °C/s). For the GC oven, a 2 min isothermal period at 70 °C increasing to 140 °C (held 2 min, rate 12 °C/min) was followed by an increase to 320 °C (held 15 min, rate 3 °C/min). The high-temperature conversion (HTC) oven was maintained at 1420 °C for all samples. Values were normalised to the Vienna Standard Mean Ocean Water (VSMOW) scale using an *n*-alkane Schimmelmann type A6 mixture (nC_16_ to nC_31_) of known isotopic composition (Arndt Schimmelmann; Biogeochemical Laboratories, Indiana University). Reproducibility greater than ±5.0‰ was attained for most samples, where reproducibility was less than ±5.0‰, this is clearly stated.

## 5. Conclusions

Here we investigated the hydrogen isotope composition of thermally unaltered and charred *Celtis australis* leaves, xylem, and bark during short-term combustions (1 h) under limited oxygen conditions in a laboratory-controlled setting, at temperatures up to 450 °C. Our results demonstrate that although significant variation is δ^2^H_wax_ is observed in the xylem and bark samples, fluctuations in δ^2^H_wax_ of leaf samples are generally minimal up to 350 °C, however, at temperatures exceeding this, considerable changes in hydrogen isotopic composition occur. Our results sound a cautionary note on the application of δ^2^H_wax_ as a palaeoclimate proxy in archaeological contexts where the depositional environment is not well understood. We propose that this proxy be enhanced by a detailed characterisation of soil microstratigraphy, which can help eliminate potential biases that arise due to the mixing of charred microscopic particles (≥450 °C) in sediment samples. Nevertheless, our observations highlight the potential for δ^2^H_wax_ to record and retain palaeoclimate information at temperatures ≤350 °C and could be employed to target thermally altered archaeological combustion substrates and link climatic and archaeological datasets at a high spatial and temporal resolution.

## Figures and Tables

**Table 1:** Quantitative results for total *n*-alkane concentration, Carbon Preference Index (CPI), and Average Chain Length (ACL). Ref indicates unheated reference samples.

***Celtis* Leaves**					
	**Ref**	**150 °C**	**250 °C**	**350 °C**	**450 °C**
Total *n*-alkane (µg/g)	459	484	360	97.0	28.8
CPI_25-33_	2.7	3.3	5.6	4.3	1.1
ACL_25-33_	30.3	30.4	30.5	30.1	29.7
***Celtis* Branch Interior (Xylem)**					
	**Ref**	**150 °C**	**250 °C**	**350 °C**	**450 °C**
Total *n*-alkane (µg/g)	0.9	0.9	1.2	1.6	0.1
CPI_25-33_	1.3	1.3	1.3	1.5	-
ACL_25-33_	26.9	26.9	27.1	26.0	25.0
***Celtis* Branch Exterior (Bark)**					
	**Ref**	**150 °C**	**250 °C**	**350 °C**	**450 °C**
Total *n*-alkane (µg/g)	18.5	4.2	3.5	8.5	0.1
CPI_25-33_	3.1	3.4	1.6	1.1	-
ACL_25-33_	26.5	26.9	27.9	26.6	-

**Table 2:** Hydrogen isotope values of individual long-chain *n*-alkanes in thermally unaltered reference samples and heated samples of *Celtis australis* leaf, branch interior (xylem), and exterior (bark). The δ^2^H_wax_ values are normalised to the Vienna Standard Mean Ocean Water (VSMOW) standard. Values are expressed in per mil (‰) with one standard deviation. Ref indicates unheated reference samples and * denotes samples where reproducibility was less than ±5.0‰ following three replicates.

***Celtis* Leaves**										
	**Ref**	**σ**	**150 °C**	**σ**	**250 °C**	**σ**	**350 °C**	**σ**	**450 °C**	**σ**
**C_27_**	−106.7	1.2	−94.3	4.3	−122.1	3.2	−120.5	2.9	−73.3	2.1
**C_29_**	−130.7	1.0	−132.0	1.4	−133.9	3.7	−134.8	0.6	−99.5	1.0
**C_31_**	−128.2	0.6	−131.3	0.9	−132.8	3.0	−135.0	1.7	−87.0	6.1 *
***Celtis* Branch Interior (Xylem)**										
	**Ref**	**σ**	**150 °C**	**σ**	**250 °C**	**σ**	**350 °C**	**σ**	**450 °C**	**σ**
**C_27_**	-	-	−146.8	1.8	−165.4	2.7	-	-	-	-
**C_29_**	-	-	−188.2	3.5	−222.7	1.3	-	-	-	-
**C_31_**	−193.4	1.1	−273.7	2.4	−241.2	2.2	-	-	-	-
***Celtis* Branch Exterior (Bark)**										
	**Ref**	**σ**	**150 °C**	**σ**	**250 °C**	**σ**	**350 °C**	**σ**	**450 °C**	**σ**
**C_27_**	−167.3	0.8	−190.8	2.3	−224.3	3.0	−204.5	0.8	-	-
**C_29_**	−158.5	4.0	−167.7	1.5	−237.8	5.2 *	−207.5	4.1	-	-
**C_31_**	−151.4	1.3	−141.2	4.2	−235.2	3.3	−224.5	0.9	-	-

## Data Availability

Data is contained within the article.

## Samples Availability

Samples are not available from the authors.
